# Effect of asymmetrical heat rise/fall on the film flow of magnetohydrodynamic hybrid ferrofluid

**DOI:** 10.1038/s41598-020-63708-y

**Published:** 2020-04-21

**Authors:** Iskander Tlili, M. T. Mustafa, K. Anantha Kumar, N. Sandeep

**Affiliations:** 1 Department for Management of Science and Technology Development, Ton Duc Thang University, Ho Chi Minh City, Vietnam; 2 Faculty of Applied Sciences, Ton Duc Thang University, Ho Chi Minh City, Vietnam; 3 Department of Mathematics, Statistics and Physics, Qatar University, Doha, 2713 Qatar; 4 Department of Mathematics, Sri Venkateswara University, Tirupati, 517502 India; 5 Department of Mathematics, Central University of Karnataka, Kalaburagi, 585367 India

**Keywords:** Applied mathematics, Computational science

## Abstract

The movement of the ferrous nanoparticles is random in the base fluid, and it will be homogeneous under the enforced magnetic field. This phenomenon shows a significant impact on the energy transmission process. In view of this, we inspected the stream and energy transport in magnetohydrodynamic dissipative ferro and hybrid ferrofluids by considering an uneven heat rise/fall and radiation effects. We studied the Fe_3_O_4_ (magnetic oxide) and CoFe_2_O_4_ (cobalt iron oxide) ferrous particles embedded in H_2_O-EG (ethylene glycol) (50–50%) mixture. The flow model is converted as ODEs with suitable similarities and resolved them using the 4th order Runge-Kutta scheme. The influence of related constraints on transport phenomena examined through graphical illustrations. Simultaneous solutions explored for both ferro and hybrid ferrofluid cases. It is found that the magnetic oxide and cobalt iron oxide suspended in H_2_O-EG (ethylene glycol) (50–50%) mixture effectively reduces the heat transfer rate under specific conditions.

## Introduction

The fluid flows across a stretching surface have attained plentiful implications in the analysis of boundary layer flow owing to its great solicitations in medical, industrial, and mechanical engineering applications. Food dispensation, silicon wafer process, hot rolling, cooling of reactors, design of precious stone, tinning of wires, continuous casting of metals, paper production, making of microchips, glass industry, crystal growth, space vehicles, polymer physics are some applications of such motion in a time-dependent liquid film flow across a surface. Anderson *et al*.^1^ discussed the problem of liquid film movement of a Newtonian fluid through a strained surface. The influence of radiative heat and variable thermal conductivity on shear thickening fluid drive of a liquid film on the unsteady permeable extending superficial inspected by Khan *et al*.^2^ and Shah *et al*.^3^ It clinched that the distribution of heat and the variable temperature parameter are linearly proportional beside the surface. Idrees *et al*.^4^ studied an elucidation of the problem of an unsteady MHD thin-film flow owing to stressed sheet in the appearance of mutable viscosity. Recently, Kumar *et al*.^5^ bestowed dual elucidations for micropolar conducting fluid flow past an overextended sheet in the presence of second-order velocity slip.

The frictional heat mechanism has an active role in industrial and scientific implications such as electric coffee fabricators, rock forms, cuisine diet, and sanitization. Chen^6^ considered a problem of an unsteady two-dimensional shear-thickening film flow over an elongated geometry to examine the influence of viscous dissipation. Later, Chen^7^ extended the problem with Joule’s heat and gave a result with the support of the numeric Keller-Box scheme. The effect of frictional heat on the natural convective 2D drive of hybrid nano liquid over a circular tube was discussed by Suresh *et al*.^8^ Numerical scrutiny was accompanied by Ramandevi *et al*.^9^ for the comparison of the heat transfer mechanism in both viscoelastic and Casson fluid flow using new heat flux under the action of and viscous dissipation. Recently, the authors^10^ discussed the impact of Joule heating on fluid motion owing to the extending sheet in the occurrence of Biot number.

Newly, scientists and researchers made an immense effort to examine the significance and separation of hybrid ferrofluids due to their applications in many branches of natural, engineering, and physical sciences. A magnetic colloid is well-known as a ferrofluid. A ferrofluid is a colloidal interruption of single-domain ferromagnetic elements in a base liquid. It has several medicinal and biological solicitations like amplifiers, revolving shaft seals, vacuum chambers, computer drives, dissipation of radiation, medicine delivery, cell parting, etc. A nanofluid consists of a single nanomaterial, whereas the hybrid nanofluid consists of more than one unlike nanoparticles with ordinary fluid. The determination behind the invention of a mixture of ferrofluids is to knob the transport phenomena. Madhesh and Kalaiselvam^11^ discussed the rheological appearances and temperature transmission of hybrid nanofluids. The magnetohydrodynamic time-dependent liquid film flow of grapheme nanoparticles embedded nanofluid under various thermal transport aspects can be viewed in ref. ^12^ The influence of drag force on the flow over an expanding surface was discussed by Sheikholeslami *et al*.^13^ Later on, the researchers^14,15^ investigated the transport phenomena of Newtonian and non-Newtonian hybrid nanoliquids under various physical effects. Kumar *et al*.^16^ discussed the effect of Brownian moment on bio convective stagnated motion of nanoliquid and presented dual solutions. It was clinched that the measure of thermal transport is high in the case of hybrid nanofluid when matched with another liquid. Sandeep^17^ deliberated the influence of drag force and variable viscosity on the hybrid nanofluid drive of liquid film over a fraught sheet.

The inspiration for radiating heat on convective movements shows a massive role in many engineering and scientific procedures like space technology, paper bowls production, freezing of metal bits, satellites, design of electronic chips, and fuel wells. The radiation is either or nonlinear depends on temperature ratio parameter values. The mechanism of asymmetrical heat rise or fall has well-known uses in drug industries and numerous manufacturing happenings like freezing of metal strips and crude oil recovery, etc. Devi and Devi^18^ considered a problem to investigate a numerical explanation for MHD flow of nanoliquid across a penetrable sheet with heat rise/fall. Kandelousi and Ellahi^19^ scrutinized a time-independent, two-dimensional ferrohydrodynamic flow through a square cavity in the occurrence of drag force. The well-known Lattice Boltzmann technique is utilized to resolve the equations of motion. Afrand *et al*.^20^ contemplated a problem to examine the Power-law fluid. The stimulus of radiation on the hydrodynamic drive of shear thickening over a stretched surface was scrutinized by Ramzan *et al*.^21^ Ghadikolaei^22^ deliberated the magnetohydrodynamic free convective motion of two different hybrid nanofluids across an overextended surface. The impact of thermic heat on MHD flow of micropolar shear-thickening nanoliquid thru a nonlinear stretched sheet was examined by Lu *et al*.^23^ numerically with the help of the Matlab package. Kumar *et al*.^24^ discussed the inspiration of asymmetrical heat generation or absorption and nonlinear thermic heat on slanting stagnated motion of shear thickening fluid across a vertical sheet in conducting field. The impact of non-linear thermal radiation and resistive heating on MHD shear-thickening nanoliquid flow over an inclined penetrable stretched sheet was reported by Ghadikolaei *et al*.^25^ Discrete heating effect on free convection flow past a vertical annulus was studied by the researchers^26–29^. Sankar *et al*.^30^ performed a numerical investigation to analyze the heat and mass transfer rates in the presence of discrete heat source.

In all the afford studies, scientists scrutinized the transport phenomena of nanoliquids past a solid geometry with numerous physical aspects. The main motto of this exploration is to provide a numerical examination of the stream and energy transport in magnetohydrodynamic dissipative ferro and hybrid ferrofluids by considering an uneven heat rise/fall and radiation effects. We studied the Fe_3_O_4_ (magnetic oxide) and CoFe_2_O_4_ (cobalt iron oxide) ferrous particles embedded in H_2_O-EG (ethylene glycol) (50–50%) mixture. The flow model is converted as ODEs with suitable similarities and resolved them using the 4th order Runge-Kutta scheme. The influence of related constraints on transport phenomena examined through graphical illustrations. Simultaneous solutions are explored for both ferrous and hybrid ferrofluid cases.

## Modelling

**Figure 1:**
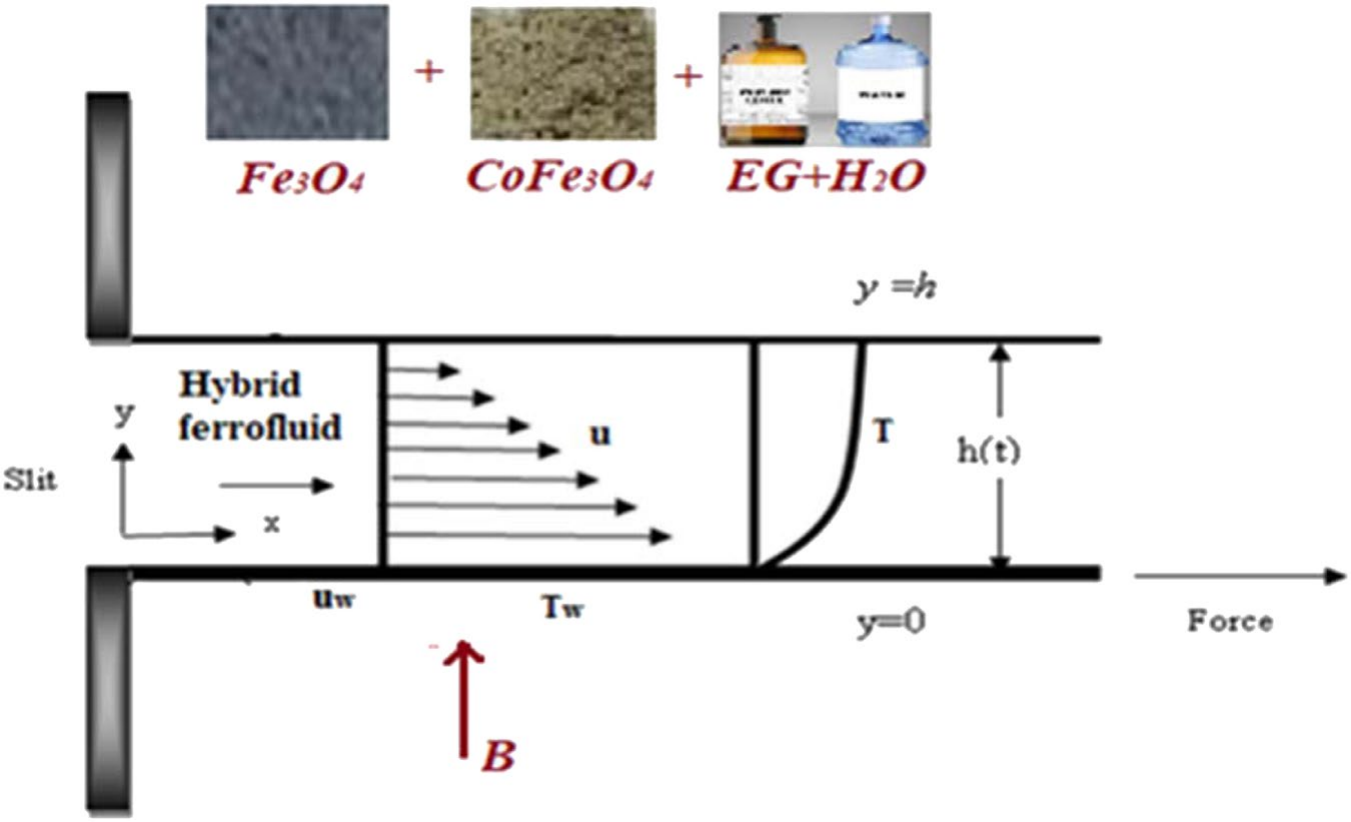
Flow configuration.

Consider a time dependent flow of EG-H_2_O based magnetic and cobalt iron oxide mixture hybrid ferrofluid past an extending sheet as depicted in Fig. 1. Here, the surface is placed lengthways *x*-axis with the temperature and momentum respectively taken as ${T}_{w}={T}_{0}-{T}_{re}\sqrt{{b}^{2}{x}^{4}/4{v}_{f}^{2}(1-\alpha t)},\,0\le {T}_{re}\le {T}_{0},$ ${\rm{and}}\,{u}_{w}=bx/(1-\alpha t)$ and $y$- axis is normal to it (where $b,\alpha$ are constants and ${T}_{0},{T}_{re}$ are slit, reference temperatures)

An electromagnetic field of forte $B={B}_{0}\sqrt{1/(1-\alpha t)}$ is measured normal to the drive. Thermal radiation, asymmetrical heat rise/fall and viscous dissipation possessions are engaged. The slip between the particles is neglected. With the above conventions, the leading transport equalities are as follows:

1 $$\frac{\partial u}{\partial x}+\frac{\partial v}{\partial y}=0,$$

2 $$u\frac{\partial u}{\partial x}+v\frac{\partial u}{\partial y}+\frac{\partial u}{\partial t}=\frac{{\mu }_{hnf}}{{\rho }_{hnf}}\frac{{\partial }^{2}u}{\partial {y}^{2}}+\frac{{\sigma }_{hnf}}{{\rho }_{hnf}}{B}^{2}u,$$

3 $$u\frac{\partial T}{\partial x}+v\frac{\partial T}{\partial y}+\frac{\partial T}{\partial t}=\frac{{k}_{hnf}}{{(\rho {c}_{p})}_{hnf}}\frac{{\partial }^{2}T}{\partial {y}^{2}}+\frac{1}{{(\rho {c}_{p})}_{hnf}}\frac{16{T}_{\infty }^{3}{\sigma }^{\ast }}{3{k}^{\ast }}\frac{{\partial }^{2}T}{\partial {y}^{2}}+{\mu }_{hnf}{\left(\frac{\partial u}{\partial y}\right)}^{2}+q\text{'}\text{'}\text{'},$$

Frontier limitations:

4 $$\begin{array}{c}u(0)={u}_{w},\,T(0)={T}_{w},v(0)=0,\,{\rm{and}}\\ {u}_{y}(h)={T}_{y}(h)=0\,,\,v={h}_{t}\,at\,y=h(t),\end{array}$$

where

5 $$q\text{'}\text{'}\text{'}=\frac{{k}_{f}b}{(1-\alpha t){v}_{f}}({A}^{\ast }({T}_{w}-{T}_{0})f\text{'}+(T-{T}_{0}){B}^{\ast }),$$

where, the dynamic viscosity, density, electrical conductivity, thermal conductivity, specific heat capacitance, Stefan–Boltzmann constant, uneven heat source/sink parameters and radiative absorption coefficient are respectively given by $\mu ,\rho ,\sigma ,k,{c}_{p},{\sigma }^{\ast },{A}^{\ast },{B}^{\ast }\,and\,{k}^{\ast }$. Here, the suffix $nf\,{\rm{and}}\,hnf$ denotes the nano and hybrid nanoliquid.

Similarity transmutations^26^

6 $$\begin{array}{c}\eta =\sqrt{\frac{b{y}^{2}}{{\beta }^{2}{v}_{f}(1-\alpha t)}},\,\psi =\sqrt{\frac{{\beta }^{2}{v}_{f}b{x}^{2}}{(1-\alpha t)}}f(\eta ),\,\theta =\frac{(T-{T}_{0})}{({T}_{w}-{T}_{0})},\\ u={\psi }_{y},v=-\,{\psi }_{x},{T}_{w}={T}_{0}-{T}_{re}\sqrt{{b}^{2}{x}^{4}/4{v}_{f}^{2}{(1-\alpha t)}^{3}}\theta (\eta ),\end{array}\}$$

The ferrofluid restrictions are taken as

7 $$\begin{array}{c}\frac{{k}_{hnf}}{{k}_{f}}=\frac{{k}_{2s}+2{k}_{f}-2{\phi }_{2}({k}_{f}-{k}_{2s})}{{k}_{2s}+2{k}_{f}+{\phi }_{2}({k}_{f}-{k}_{2s})}\times {k}_{nf},{k}_{nf}=\frac{{k}_{1s}+2{k}_{f}-2{\phi }_{1}({k}_{f}-{k}_{1s})}{{k}_{1s}+2{k}_{f}+{\phi }_{1}({k}_{f}-{k}_{1s})},\\ \frac{{\rho }_{hnf}}{{\rho }_{f}}=(1-{\phi }_{2})\left[(1-{\phi }_{1})+\frac{{\phi }_{1}{\rho }_{1s}}{{\rho }_{f}}\right]+\frac{{\phi }_{2}{\rho }_{2s}}{{\rho }_{f}},\frac{{\mu }_{hnf}}{{\mu }_{f}}=\frac{1}{{(1-{\phi }_{1})}^{2.5}{(1-{\phi }_{2})}^{2.5}},\\ \frac{{(\rho {c}_{p})}_{hnf}}{{(\rho {c}_{p})}_{f}}=(1-{\phi }_{2})\left[(1-{\phi }_{1})+\frac{{\phi }_{1}{(\rho {c}_{p})}_{1s}}{{(\rho {c}_{p})}_{f}}\right]+\frac{{\phi }_{2}{(\rho {c}_{p})}_{2s}}{{(\rho {c}_{p})}_{f}},\\ \frac{{\sigma }_{hnf}}{{\sigma }_{f}}=\left[1+\frac{3({\sigma }_{1s}{\phi }_{1s}-\phi {\sigma }_{f})+{\phi }_{2s}{\sigma }_{2s}}{{\sigma }_{1s}(1-{\phi }_{1s})+{\sigma }_{2s}(1-{\phi }_{2s})+(2+\phi ){\sigma }_{f}}\right],\phi ={\phi }_{1}+{\phi }_{2},\end{array}\}$$

Using Eqs. (5) to (7), the Eqs. (2) to (4) can be distorted as

8 $$\begin{array}{c}\frac{1}{{(1-{\phi }_{1})}^{2.5}{(1-{\phi }_{2})}^{2.5}}f\text{'}\text{'}\text{'}+\left\{(1-{\phi }_{2})\left((1-{\phi }_{1})+\frac{{\phi }_{1}{\rho }_{1s}}{{\rho }_{f}}\right)+\frac{{\phi }_{2}{\rho }_{2s}}{{\rho }_{f}}\right\}\\ \times \lambda (f\text{'}\text{'}-Sf\text{'}-f{\text{'}}^{2}-0.5S\eta f\text{'}\text{'})\\ \,-M\left(1+\frac{3({\sigma }_{1s}{\phi }_{1s}-\phi {\sigma }_{f})+{\phi }_{2s}{\sigma }_{2s}}{{\sigma }_{1s}(1-{\phi }_{1s})+{\sigma }_{2s}(1-{\phi }_{2s})+(2+\phi ){\sigma }_{f}}\right)f\text{'}=0,\end{array}\}$$

9 $$\begin{array}{c}\left(\frac{{k}_{2s}+2{k}_{f}-2{\phi }_{2}({k}_{f}-{k}_{2s})}{{k}_{2s}+2{k}_{f}+{\phi }_{2}({k}_{f}-{k}_{2s})}\times {k}_{nf}+\frac{4}{3}R\right)\theta \text{'}\text{'}+\Pr Ecf\text{'}{\text{'}}^{2}+{B}^{\ast }\theta +{A}^{\ast }f\text{'}\\ -\Pr \left((1-{\phi }_{2})\left((1-{\phi }_{1})+\frac{{\phi }_{1}{(\rho {c}_{p})}_{1s}}{{(\rho {c}_{p})}_{f}}\right)+\frac{{\phi }_{2}{(\rho {c}_{p})}_{2s}}{{(\rho {c}_{p})}_{f}}\right)\lambda (2f\text{'}\theta 0.5S(3\theta +\eta \theta \text{'})-f\theta \text{'})=0,\end{array}\}$$

Transformed boundaries:

10 $$\begin{array}{c}f(\eta )=0,\theta (\eta )=1,f\text{'}(\eta )=1,\,at\,\eta =0,\,\\ f(1)=0.5S,{\frac{{\partial }^{2}f}{\partial {\eta }^{2}}|}_{\eta =1}=0,{\frac{\partial \theta }{\partial \eta }|}_{\eta =1}=0,\end{array}$$

where, ${\phi }_{1},{\phi }_{2}$ denotes the nanoparticle volume fraction and the suffixes $f,s$ denotes the fluid and solid particles. And the dimensionless magnetic field parameter, Prandtl number, Radiation parameter, Unsteadiness parameter, Eckert number and film thickness quantities are specified by

11 $$M=\frac{{\sigma }_{f}{B}_{0}^{2}}{b{\rho }_{f}},\Pr =\frac{{v}_{f}}{{\alpha }_{f}},R=\frac{4{\sigma }^{\ast }{T}_{0}^{3}}{{k}^{\ast }{k}_{f}},S=\frac{\alpha }{b},Ec=\frac{{u}_{w}^{2}}{{({c}_{p})}_{f}({T}_{w}-{T}_{0})},\lambda ={\beta }^{2},$$

The reduced Nusselt number $N{u}_{x}$ is specified as

12 $$N{u}_{x}=-{\beta }^{-1}{\mathrm{Re}}_{x}^{1/2}\left(\frac{{k}_{2s}+2{k}_{f}-2{\phi }_{2}({k}_{f}-{k}_{2s})}{{k}_{2s}+2{k}_{f}+{\phi }_{2}({k}_{f}-{k}_{2s})}\times {k}_{nf}+1.33R\right)\theta \text{'}(0),$$

with the Reynolds number ${\mathrm{Re}}_{x}=\frac{{u}_{w}x}{{v}_{f}}$.

## Results and Discussion

The Eqs. (8) and (9) with the constraints in Eq. (10) are resolved by the RK-shooting scheme. The assets of non-dimensional restrictions on the drive, energy fields along with Nusselt number are elucidated explicitly. The results are displayed for (H_2_O-EG-Fe_3_O_4_) water-EG-magnetic oxide ferrofluid (solid lines), (H_2_O-EG-CoFe_2_O_4_) water-EG-cobalt iron oxide ferrofluid (dashed lines) and water-EG- magnetic/cobalt iron oxide hybrid ferrofluid (dotted lines) cases. For computing purpose, the dimensionless quantities can be considered as $M=3,R=1,{\phi }_{1}={\phi }_{2}=0.1;Ec=S=\lambda ={A}^{\ast }={B}^{\ast }=0.5,\Pr =30.$ Table 1 predicts the physical properties, and Table 2 discusses the authentication of the outputs.

**Table 1:** Thermophysical possessions.

Property	*ρ*(Kg/m^3^)	*c*_*p*_(J/KgK)	*k*(W/mK)	*σ*(S/m)
${{\rm{H}}}_{2}{\rm{O}}+{\rm{EG}}(50 \% -50 \% )$	1057	3287	0.424	0.005
Magnetic oxide	5180	670	9.8	0.74×10^6^
Cobalt iron oxide	4908	700	3.6	1.1×10^7^

**Table 2:** Authentication of the outputs of $-\theta \text{'}(0)$ when $\Pr =1,\,M=\phi =Ec=R=0$.

*S*	Xu *et al*.^26^	Sandeep *et al*.^12^	Current Results
1.0	2.67722	2.67722	2.677224531
1.2	1.99959	1.99959	1.999593210
1.4	1.44775	1.44775	1.447752761
1.6	0.95669	0.95669	0.956693254

**Figure 2:**
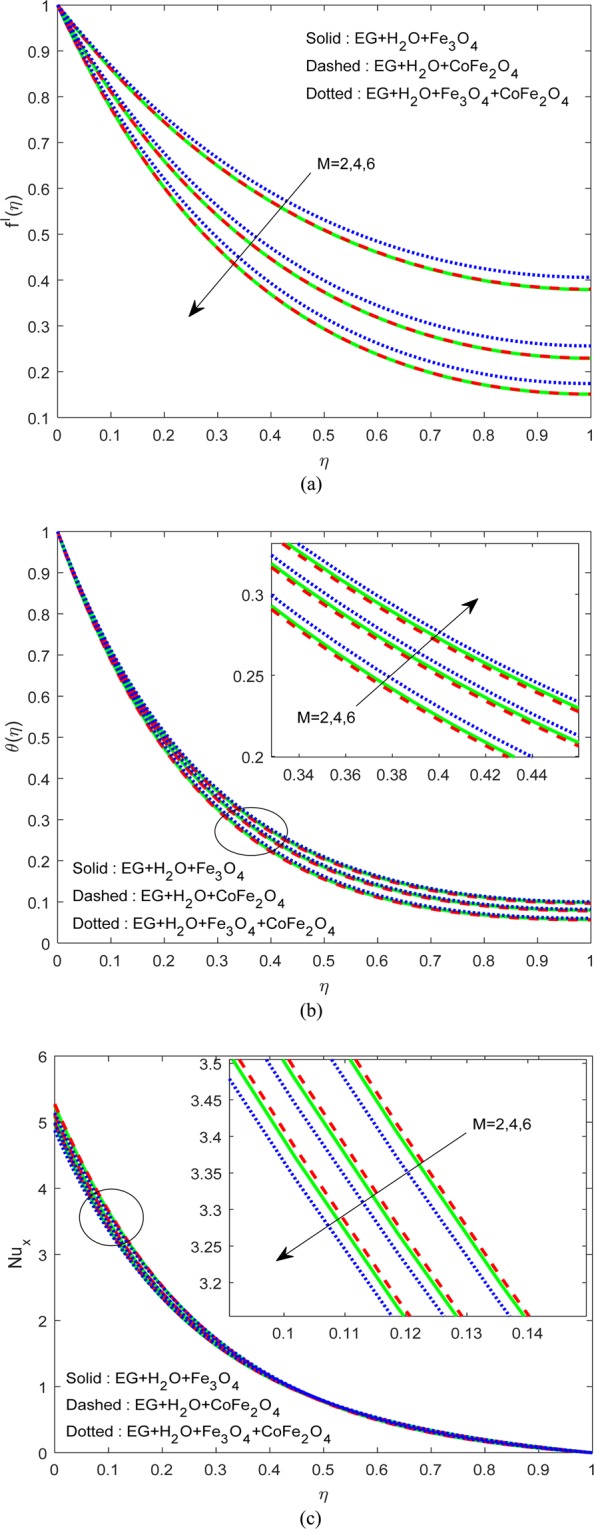
Variation of $M$ on (**a**) Flow (**b**) Energy (**c**) $N{u}_{x}$.

Figure 2 explains the impression of $M$ on the drive and thermal fields along with Nusselt number. The momentum boundary layer of water-EG-magnetic/cobalt iron oxide hybrid ferrofluid is less influenced by $M$ when equated with water-EG-magnetic oxide and water-EG-cobalt iron oxide ferrofluids. And the opposite trend has been noticed in the thermal boundary layer as displayed in Fig. 2(a). The reason for these trends is the drag force developed against the flow due to the imposing of external magnetic force. As a rise in the resultant temperature, we noticed a fall in the Nusselt number, as shown in Fig. 2(c). Notably, it is less in H_2_O-EG-Fe_3_O_4_/CoFe_2_O_4_ hybrid ferrofluid when compared to other two ferrofluids.

**Figure 3:**
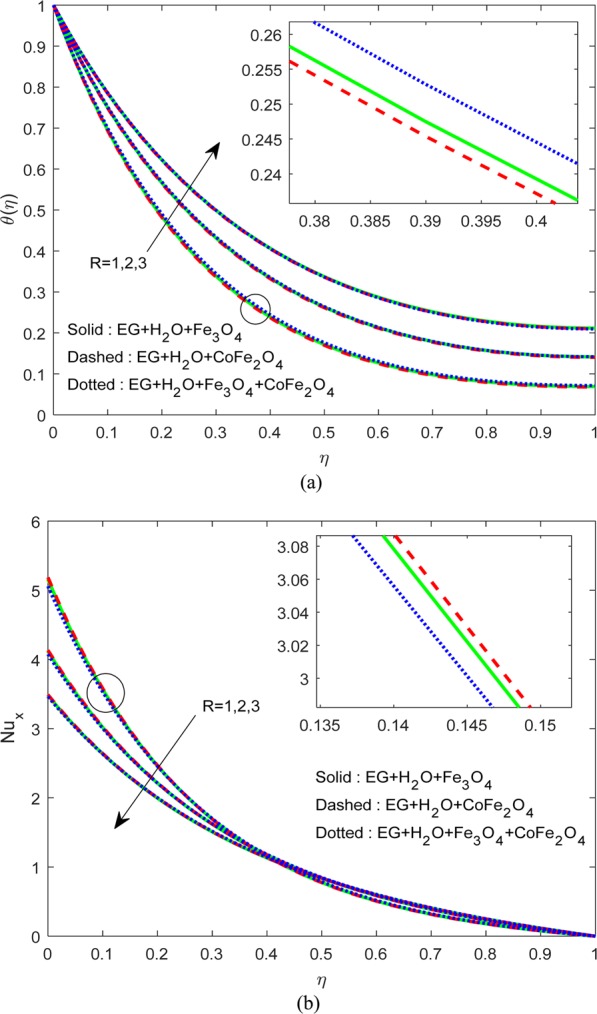
Variation of $R$ on (**a**) Flow (**b**) $N{u}_{x}$.

**Figure 4:**
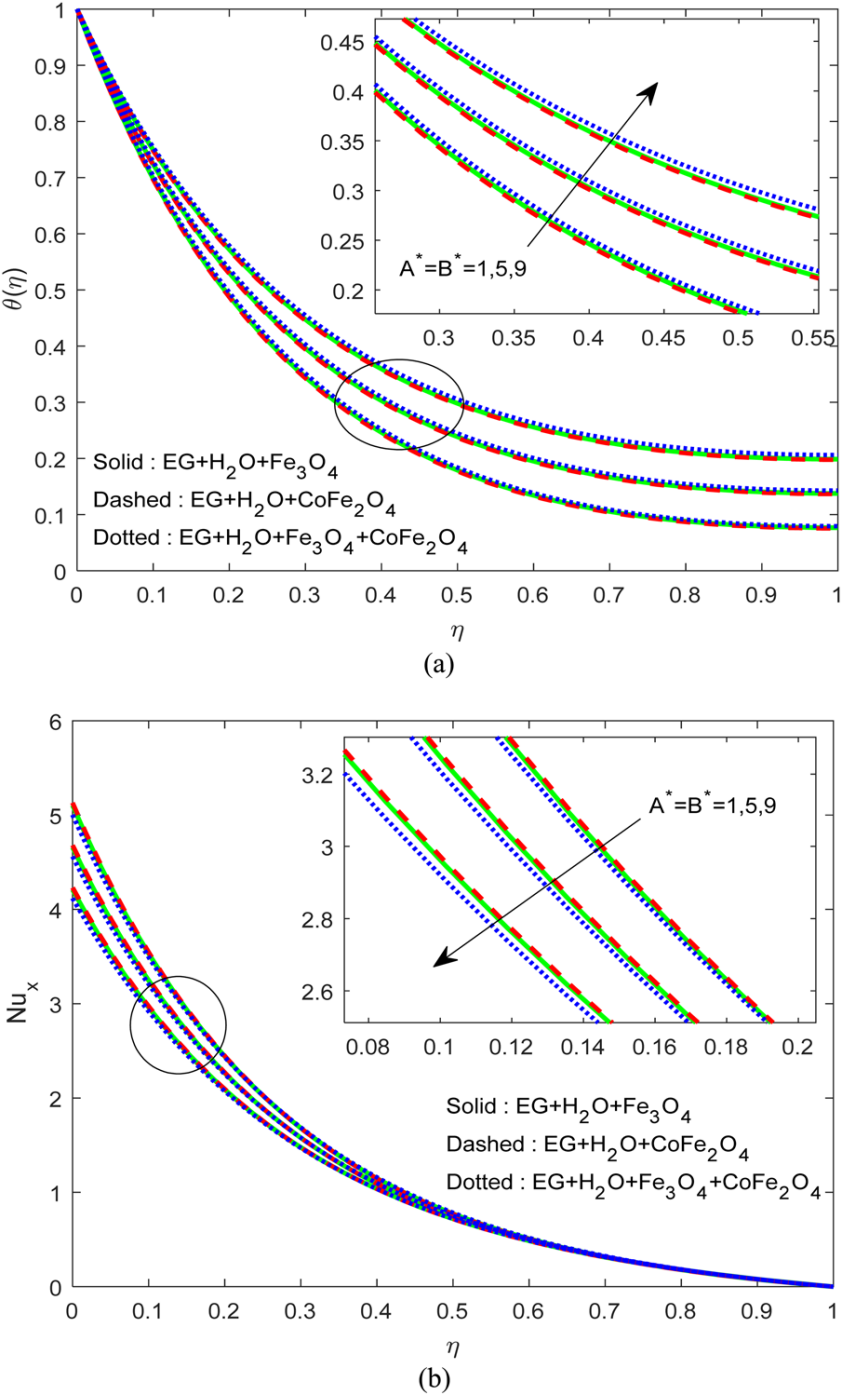
Variation of ${A}^{\ast }\,\& \,{B}^{\ast }$ on (**a**) flow (**b**) $N{u}_{x}$.

Figure 3 explicate the influence of $R$ and uneven heat rise/fall on $\theta (\eta )\,{\rm{and}}\,N{u}_{x}$. As per the general nature of $R,\,{A}^{\ast }\,{\rm{and}}\,{B}^{\ast }$, a rise in the heat input primes to upsurge in the heat field. A similar drift was followed by Fig. 4. However, we observed a significant hike in the thermal field of H_2_O-EG-Fe_3_O_4_/CoFe_2_O_4_ hybrid ferrofluid when compared to the other two ferrofluids. These may be due to the enhanced heat conduction between the Fe_3_O_4_-CoFe_2_O_4_ solid particles. As a result, we found decay in $N{u}_{x}$ in all cases for growing numbers of $R,\,{A}^{\ast }\,{\rm{and}}\,{B}^{\ast }$ (see Figs. 3(b) and 4(b)).

**Figure 5:**
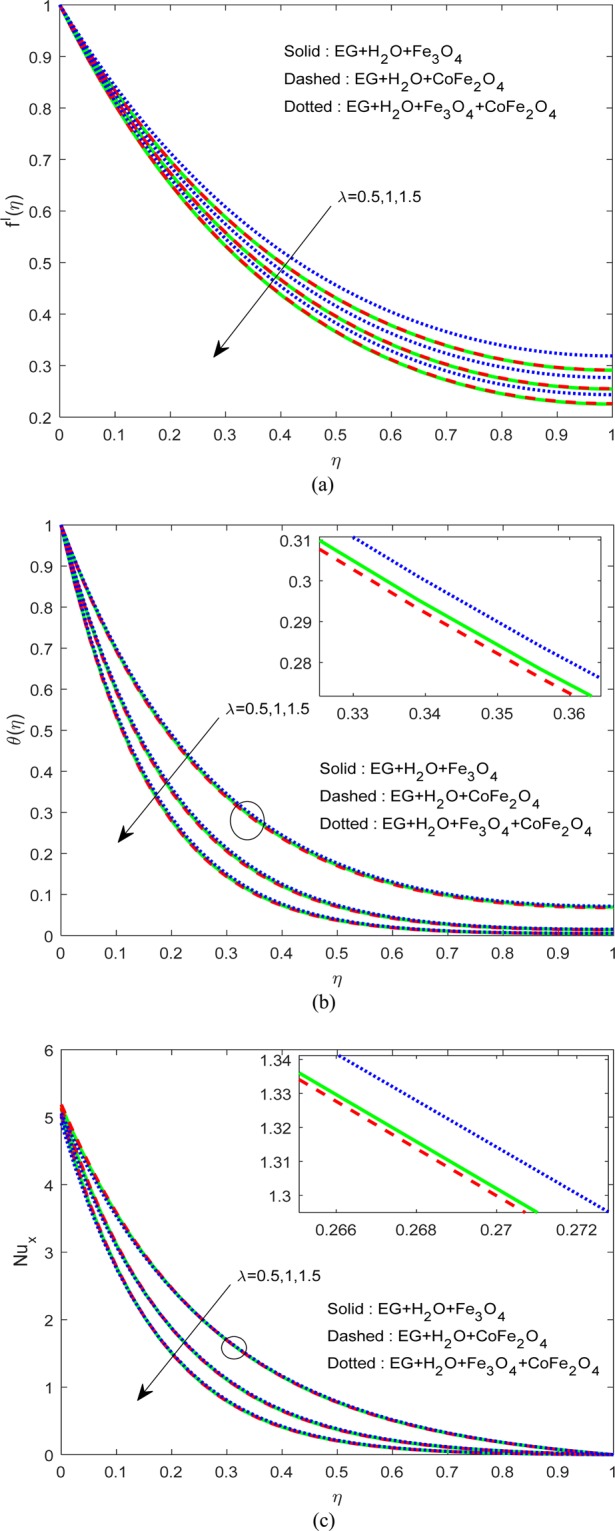
Variation of $\lambda$ on (**a**) Flow (**b**) Energy (**c**) $N{u}_{x}$.

Figure 5 expounds the result of $\lambda$ on $f\text{'}(\eta ),\theta (\eta )\,{\rm{and}}\,N{u}_{x}$. We perceived a depreciation in momentum and thermal arenas for boosting values of $\lambda$. Notably, we noticed that the impact of $\lambda$ is high on the thermal filed of H_2_O-EG-CoFe_2_O_4_ ferrofluid when equated to water-EG-magnetic oxide ferrofluid and water-EG-magnetic/cobalt iron oxide hybrid ferrofluid. Generally, a rise in the film thickness leads to enlarging the flow filed and hence reduces the heat transfer. Cobalt may be the reason for the additional reduction in the heat transfer of H_2_O-EG-CoFe_2_O_4_ ferrofluid.

**Figure 6:**
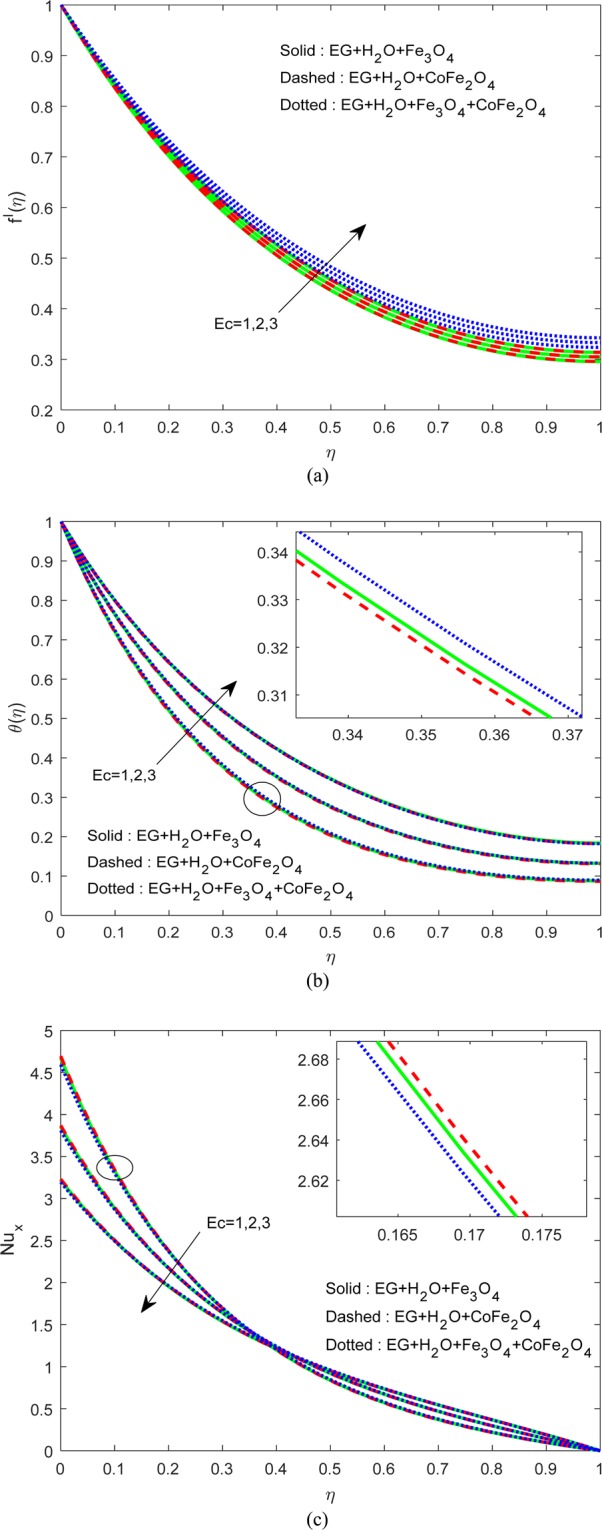
Variation of $Ec$ on (**a**) Flow (**b**) Energy (**c**) $N{u}_{x}$.

**Figure 7:**
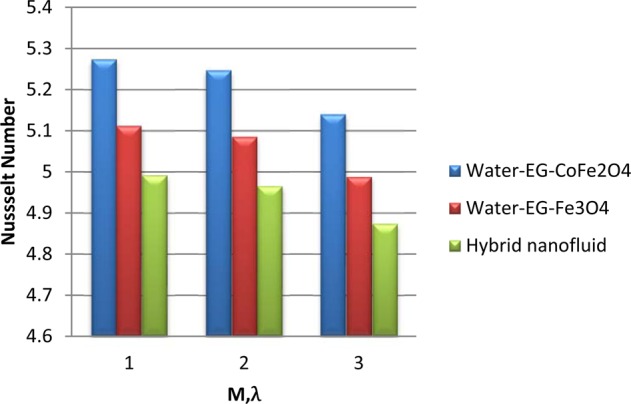
Variation of $M,\lambda$ on $N{u}_{x}$.

**Figure 8:**
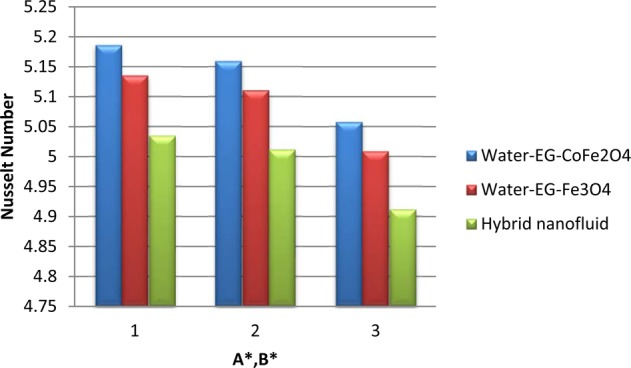
Variation of ${A}^{\ast },{B}^{\ast }$ on $N{u}_{x}$.

Figure 6 illustrate the control of viscous dissipation on $f\text{'}(\eta ),\theta (\eta )\,{\rm{and}}\,N{u}_{x}$. It is profound that the growing values of $Ec$ effectively escalate the thermal and drive boundary layers. Physically, drive force can be converted as heat energy in viscous fluids. If the viscosity of the fluid is high, then more internal heat energy will generate and hence the heat transfer. Interestingly, enhancing the fluid viscosity commendably enhancing the flow and thermal fields of H_2_O-EG-Fe_3_O_4_/CoFe_2_O_4_ hybrid ferrofluid when equated to the other two ferrofluids. Figures 7 and 8 exemplify the impression of $M,\lambda ,\,{A}^{\ast }\,{\rm{and}}\,{B}^{\ast }$ on Nusselt number. It is evident that the boosting standards of $M,\lambda ,\,{A}^{\ast }\,{\rm{and}}\,{B}^{\ast }$ decline the heat transfer rate in all cases. Mainly, this influence is high on H_2_O-EG-Fe_3_O_4_/CoFe_2_O_4_ hybrid ferrofluid.

## Conclusions

Due to the numerous applications of ferrofluid fluids in the heat transfer process, a computational analysis is performed to investigate the drive and heat transport in EG-H2O/Fe3O4 ferroliquid, water-EG/CoFe2O4 ferroliquid and water-EG-magnetic oxide/cobalt iron oxide ferrofluid hybrid ferrofluid in the presence of thermal radiation, dissipation and uneven energy generation/fall. Observations of the current analysis are listed below:

- The drive of water-EG-magnetic oxide/cobalt iron oxide hybrid ferrofluid is effectively controlled by $M$.
- Thermal radiation and asymmetrical heat rise/fall regulates the energy transport in
  H_2_O-EG-Fe_3_O_4_/CoFe_2_O_4_ hybrid ferrofluid.
- Enhancement in film thickness and addition of cobalt leads to a gradual decline in the thermal boundary layer thickness.
- CoFe_2_O_4_ may works like an insulator by increasing the concentration of cobalt.
- H_2_O-EG-Fe_3_O_4_/CoFe_2_O_4_ hybrid ferrofluid may be used as a coolant by balancing the cobalt levels.
- By monitoring the viscous levels, we can control the internal heat transfer of H_2_O-EG-Fe_3_O_4_/CoFe_2_O_4_ hybrid ferrofluid.
